# Supplementary material for: Ordered patterning of the sensory system is susceptible to stochastic features of gene expression
Source: eLife. 2020 Feb 26;9:e53638. doi: 10.7554/eLife.53638 (PMC7064346; doi:10.7554/eLife.53638)
Supplement: Transparent reporting form [file elife-53638-transrepform.pdf]

eLIFE
1st Floor
24 Hills Road
Cambridge CB2 1JP, UK
P 01223 855340
W elifesciences.org
T @elife

eLife's transparent reporting form

We encourage authors to provide detailed information *within their submission* to facilitate the interpretation and replication of experiments. Authors can upload supporting documentation to indicate the use of appropriate reporting guidelines for health-related research (see EQUATOR Network), life science research (see the BioSharing Information Resource), or the ARRIVE guidelines for reporting work involving animal research. Where applicable, authors should refer to any relevant reporting standards documents in this form.

If you have any questions, please consult our Journal Policies and/or contact us: editorial@elifesciences.org.

Sample-size estimation

- You should state whether an appropriate sample size was computed when the study was being designed
- You should state the statistical method of sample size computation and any required assumptions
- If no explicit power analysis was used, you should describe how you decided what sample (replicate) size (number) to use

Please outline where this information can be found within the submission (e.g., sections or figure legends), or explain why this information doesn't apply to your submission:

Sample size for all experiments is listed under 'Quantification and Statistical Analysis' in methods. For Fano factor and average protein, point-estimates and 95% confidence intervals were estimated by bootstrap technical replication. For all such point-estimates, adequate sample size was confirmed by normal distribution of bootstrapped replicates. For adult wing analysis, chemosensory bristle density distributions were checked for normality. For ectopic mechanosensory bristle analysis, sample sizes were computed for power 0.8 after a pilot study. To compare *sens* alleles between 57F5 vs 22A3, appropriate sample size was determined to be n ≥ 40. To statistically distinguish an effect of miR-9a for *sens* alleles at 22A3 with power 0.8, required sample size was estimated at n >34000 from pilot data. Since, this indicated a negligible effect, data were only gathered for n ≥ 60 for all genotypes.

Replicates

- You should report how often each experiment was performed
- You should include a definition of biological versus technical replication
- The data obtained should be provided and sufficient information should be provided to indicate the number of independent biological and/or technical replicates
- If you encountered any outliers, you should describe how these were handled
- Criteria for exclusion/inclusion of data should be clearly stated
- High-throughput sequence data should be uploaded before submission, with a private link for reviewers provided (these are available from both GEO and ArrayExpress)

Please outline where this information can be found within the submission (e.g., sections or figure legends), or explain why this information doesn't apply to your submission:

Sample size for all experiments is listed under 'Quantification and Statistical Analysis' in methods. For single cell data, the pixel area count of each segmented cell was considered a technical replicate. Cells with at least n = 100 pixels were carried forward for expression analysis. Every cell in the sample is considered a biological replicate. Since ~1000 cells could be measured in one wing disc, experiments utilized 12-100 wing disc replicates per genotype. For Fano factor and average protein, point-estimates and 95% confidence intervals were estimated by bootstrap technical replication. For all such point-estimates, adequate sample size was confirmed by normal distribution of bootstrapped replicates. To estimate Sens protein concentrations in live wing disc cells, cells were grouped by proximity to an S-cell. Each cell was considered a biological replicate. FCS measurements were performed in multiple volumes in the same nucleus and averaged (technical replicate). Measurements with marked bleaching, counts per molecule below 0.5 kHz were excluded. For qPCR and ELISA analysis, samples were analyzed in technical triplicates. Each time-point used to estimate the decay constant is considered a biological replicate. Within each time-point, data were gathered for three biological replicate groups of 5-10 discs each. For adult wing analysis, each wing is considered a biological replicate. For chemosensory bristle density, each pair of bristle neighbors is a biological replicate. There was no technical replication.

eLife Sciences Publications, Ltd is a limited liability non-profit non-stock corporation incorporated in the State of Delaware, USA, with company number 5030732, and is registered in the UK with company number FC030576 and branch number BR015634 at the address 1st Floor, 24 Hills Road, Cambridge CB2 1JP | August 2014

Statistical reporting

- Statistical analysis methods should be described and justified
- Raw data should be presented in figures whenever informative to do so (typically when N per group is less than 10)
- For each experiment, you should identify the statistical tests used, exact values of N, definitions of center, methods of multiple test correction, and dispersion and precision measures (e.g., mean, median, SD, SEM, confidence intervals; and, for the major substantive results, a measure of effect size (e.g., Pearson's r, Cohen's d)
- Report exact p-values wherever possible alongside the summary statistics and 95% confidence intervals. These should be reported for all key questions and not only when the p-value is less than 0.05.

Please outline where this information can be found within the submission (e.g., sections or figure legends), or explain why this information doesn't apply to your submission:

Source data is provided in the data package uploaded on github. Calculation of all point-estimates and 95% confidence intervals is described in the methods section and/or relevant figure legends. Sample sizes are listed under 'Quantification and Statistical Analysis'. P-values and R^2 values are provided in figures and figure supplements. Odds ratio analysis reported in Figure 7 - source data 1. Where possible, data are plotted to show 95% confidence intervals of fit or point-estimate.

(For large datasets, or papers with a very large number of statistical tests, you may upload a single table file with tests, Ns, etc., with reference to sections in the manuscript.)

Group allocation

- Indicate how samples were allocated into experimental groups (in the case of clinical studies, please specify allocation to treatment method); if randomization was used, please also state if restricted randomization was applied
- Indicate if masking was used during group allocation, data collection and/or data analysis

Please outline where this information can be found within the submission (e.g., sections or figure legends), or explain why this information doesn't apply to your submission:

Groups were allocated according to genotype. Data were gathered in the same experimental run for multiple genotypes. For image data, all samples were masked during data collection. Segmentation, image processing and expression analysis were carried out for all groups identically with a computational pipeline. Sample labels were masked in all steps. For adult wing analysis, data were masked during imaging and bristle counting. For mRNA or protein decay analysis, data were grouped by time point.

eLife Sciences Publications, Ltd is a limited liability non-profit non-stock corporation incorporated in the State of Delaware, USA, with company number 5030732, and is registered in the UK with company number FC030576 and branch number BR015634 at the address 1st Floor, 24 Hills Road, Cambridge CB2 1JP | August 2014

eLIFE
1st Floor
24 Hills Road
Cambridge CB2 1JP, UK
P 01223 855340
W elifesciences.org
T @elife

Additional data files ("source data")

- We encourage you to upload relevant additional data files, such as numerical data that are represented as a graph in a figure, or as a summary table
- Where provided, these should be in the most useful format, and they can be uploaded as "Source data" files linked to a main figure or table
- Include model definition files including the full list of parameters used
- Include code used for data analysis (e.g., R, MatLab)
- Avoid stating that data files are "available upon request"

Please indicate the figures or tables for which source data files have been provided:

Experimental data is provided for all main Figures 1-7, Figure 3 – figure supplement 2 and Figure 7 – figure supplements 1-3. R script for two-color expression analysis and MATLAB scripts for cell segmentation, model parameters, and model simulations are provided at https://github.com/ritika-giri/stochastic-noise. All data and code are available at https://github.com/ritika-giri/stochastic-noise.

Datasets of published HiC analysis of the Drosophila genome are from Stadler, M.R., Haines, J.E., and Eisen, M.B. (2017). Convergence of topological domain boundaries, insulators, and polytene interbands revealed by high-resolution mapping of chromatin contacts in the early *Drosophila* melanogaster embryo. eLife *6*, 1–29. doi: 10.7554/eLife.29550. Data accession (GSE100370).
Dataset of DNase sensitivity on Drosophila genome are from Li, X., MacArthur, S., Bourgon, R., Nix, D., Pollard, D.A., Iyer, V.N., Hechmer, A., Simirenko, L., Stapleton, M., Hendriks, C.L.L., et al. (2008). Transcription factors bind thousands of active and inactive regions in the *Drosophila* blastoderm. PLOS Biology *6*, e27. https://doi.org/10.1371/journal.pbio.0060027.
ChIP-seq datasets of the Drosophila genome are from Nègre, N., Brown, C.D., Shah, P.K., Kheradpour, P., Morrison, C.A., Henikoff, J.G., Feng, X., Ahmad, K., Russell, S., White, R.A.H., et al. (2010). A comprehensive map of insulator elements for the *Drosophila* genome. PLOS Genetics *6*, e1000814. https://doi.org/10.1371/journal.pgen.1000814 Data accession (GSE16245)

eLife Sciences Publications, Ltd is a limited liability non-profit non-stock corporation incorporated in the State of Delaware, USA, with company number 5030732, and is registered in the UK with company number FC030576 and branch number BR015634 at the address 1st Floor, 24 Hills Road, Cambridge CB2 1JP | August 2014
